# The Institutional Treatment of Tuberculosis

**Published:** 1919-01-11

**Authors:** 


### THE INSTITUTIONAL TREATMENT OF TUBERCULOSIS.

THE increase in the incidence of pulmonary tuberculosis during the last four years and the position of the discharged tuberculous soldier have accentuated the need for a greater measure of uniformity with regard to institutional schemes for the treatment of tuberculosis. At the present time there is an obvious trend in certain quarters to advocate the provision of open-air industrial colonies for the treatment and training of able-bodied tuberculous soldiers without giving due regard to the urgent need of a much more extensive provision of hospital accommodation for acute and advanced cases of the disease. There are three forms of institutional accommodation provided for the treatment of pulmonary tuberculosis, viz., sanatorium, hospital and industrial colony, but, unfortunately, there exists no uniformity among different authorities as to the method of providing these various forms of institutional treatment. In considering the manner in which such methods of treatment can be carried out most efficiently and most economically, two facts at once become fairly obvious. The first is that without adequate hospital accommodation the provision of industrial colonies is placing the cart before the horse, and the second is that, given adequate hospital accommodation, then the difference between the sanatorium and the industrial colony becomes so slight that they can readily be merged into one so as to constitute the "industrial sanatorium."

Two institutions are therefore all that are necessary provided the accommodation is adequate. The absence of adequate hospital accommodation has lowered the standard of the sanatorium, and has so accentuated the difference between that institution and the industrial colony as to lead to the mistaken idea that a third institutional system is essential to the successful treatment of tuberculosis. To multiply institutions for the treatment of tuberculosis more than is necessary is costly, and leads to useless dissipation of effort. The danger of the separate colony system is that it will direct thought and action away from the tuberculosis hospital, which is the foundation upon which the success of the whole scheme of treatment depends. Adequate hospital accommodation and the provision of a central industrial sanatorium for each area will be found to constitute a sane and economical system of institutional treatment.

